# PBX homeobox 1 enhances hair follicle mesenchymal stem cell proliferation and reprogramming through activation of the AKT/glycogen synthase kinase signaling pathway and suppression of apoptosis

**DOI:** 10.1186/s13287-019-1382-y

**Published:** 2019-08-23

**Authors:** Yixu Jiang, Feilin Liu, Fei Zou, Yingyao Zhang, Bo Wang, Yuying Zhang, Aobo Lian, Xing Han, Zinan Liu, Xiaomei Liu, Minghua Jin, Dianliang Wang, Gang Li, Jinyu Liu

**Affiliations:** 1 The Key Laboratory of Pathobiology, Ministry of Education, Department of Pathology, College of Basic Medical Sciences, Jilin University, 126 Xinmin Avenue, Changchun, 130021 China; 2 Department of Ophthalmology, The Second Hospital of Jilin University, Changchun, 130021 China; 3 Department of Pediatrics, The First Hospital of Jilin University, Changchun, 130021 China; 4 Department of Toxicology, School of Public Health, Jilin University, 1163 Xinmin Avenue, Changchun, 130021 China; 5 Stem Cell and Tissue Engineering Research Laboratory, PLA Rocket Force Characteristic Medical Center, Beijing, 100088 China; 6 Department of Orthopaedics & Traumatology, Li Ka Shing Institute of Health Sciences, Chinese University of Hong Kong, Prince of Wales Hospital, Hong Kong, 999077 China

**Keywords:** Hair follicle mesenchymal stem cells, PBX homeobox 1, NANOG, AKT, Glycogen synthase kinase 3β, Apoptosis

## Abstract

**Background:**

PBX homeobox 1 (PBX1) is involved in the maintenance of the pluripotency of human embryonic and hematopoietic stem cells; however, the effects of PBX1 in the self-renewal and reprogramming of hair follicle mesenchymal stem cells (HF-MSCs) are unclear. The AKT/glycogen synthase kinase (GSK) 3β pathway regulates cell metabolism, proliferation, apoptosis, and reprogramming, and p16 and p21, which act downstream of this pathway, regulate cell proliferation, cell cycle, and apoptosis induced by reprogramming. Here, we aimed to elucidate the roles of PBX1 in regulating the proliferation and reprogramming of HF-MSCs.

**Methods:**

A lentiviral vector designed to carry the *PBX1* sequence or *PBX1* short hairpin RNA sequence was used to overexpress or knock down PBX1. The roles of PBX1 in proliferation and apoptosis were investigated by flow cytometry. Real-time polymerase chain reaction was performed to evaluate pluripotent gene expression. Dual-luciferase reporter assays were performed to examine the transcriptional activity of the *NANOG* promoter. Western blotting was performed to identify the molecules downstream of PBX1 involved in proliferation and reprogramming. Caspase3 activity was detected to assess HF-MSC reprogramming. The phosphatidylinositol 3-kinase/AKT inhibitor LY294002 was used to inhibit the phosphorylation and activity of AKT.

**Results:**

Overexpression of PBX1 in HF-MSCs increased the phosphorylation of AKT and nuclear translocation of β-catenin, resulting in the progression of the cell cycle from G_0_/G_1_ to S phase. Moreover, transfection with a combination of five transcription factors (SOMKP) in HF-MSCs enhanced the formation of alkaline phosphatase-stained colonies compared with that in HF-MSCs transfected with a combination of four transcription factors (SOMK). PBX1 upregulated *Nanog* transcription by activating the promoter and promoted the expression of endogenous *SOX2* and *OCT4*. Furthermore, PBX1 expression activated the AKT/glycogen synthase kinase (GSK) 3β pathway and reduced apoptosis during the early stages of reprogramming. Inhibition of phospho-AKT or knockdown of PBX1 promoted mitochondrion-mediated apoptosis and reduced reprogramming efficiency.

**Conclusions:**

PBX1 enhanced HF-MSC proliferation, and HF-MSCs induced pluripotent stem cells (iPSC) generation by activating the AKT/GSK3β signaling pathway. During the reprogramming of HF-MSCs into HF-iPSCs, PBX1 activated the *NANOG* promoter, upregulated *NANOG*, and inhibited mitochondrion-mediated apoptosis via the AKT/GSK3β pathway during the early stages of reprogramming.

## Background

Increasing evidence has shown that transcription factors (TFs) orchestrate a complicated gene expression network and synergistically interact in a temporal and spatial manner to maintain stem cell self-renewal, multipotency, and reprogramming of somatic cells into pluripotent stem cells (PSCs). Cells recapture the developmental potency by the introduction of specific TFs, reprogramming proteins, chemical compounds, microRNAs, and antibodies, indicating great potential for biomedical research and regenerative medicine [1–5]. In general, the generation of inducible PSCs (iPSCs) by transduction with SRY-box 2 (*SOX2*), octamer-binding transcription factor 4 (*OCT4*), *c-MYC*, and Kruppel-like factor 4 (*KLF4*) (SOMK) is a highly reproducible but inefficient process and maybe one of the main hurdles for the therapeutic application of iPSCs. In recent years, many researchers have focused on the identification of important players that can enhance or inhibit the reprogramming process, such as *ZIC3*, *NAC1*, and *PHLDA3* [6–8].

PBX homeobox 1 (PBX1) is a homeodomain TF that forms hetero-oligomeric complexes with HOX and transcription activator-like effector proteins to regulate numerous embryonic processes, including morphologic patterning, organogenesis, and hematopoiesis [9–11]. PBX1 is a three-amino acid loop extension homeodomain TF that dimerizes with other homeodomain proteins via a PBC domain to form nuclear complexes, which can enhance protein binding to DNA [12]. Research from Wang’s group has shown that there is a feedback interaction loop between *PBX1* and *NANOG* [13]. Moreover, PBX1 binding to the *NANOG* promoter individually or in combination with OCT4 and KLF4 activate *NANOG* transcription and subsequently support the self-renewal capability of human embryonic stem cells (hESCs) [14].

As a serine-threonine kinase, AKT regulates many downstream signaling pathways that control cell metabolism, proliferation, apoptosis, and reprogramming [15–17]. AKT phosphorylation upregulates cyclin D1 by inhibiting the expression of p16 and p21, which shift hair follicle (HF) mesenchymal stem cells (MSCs) at the G_1_ phase to the S phase [18]. Acting downstream of AKT/GSK3β signaling, p16 and p21 inhibit cyclin-dependent kinases dynamically and regulate proliferation by arresting cell cycle at G_1_/S phase. AKT activation can upregulate glucose transporters and metabolic enzymes involved in glycolysis, thereby enhancing the generation of iPSCs from human somatic cells [19, 20]. In the primate iPSC pluripotency network, the AKT pathway significantly upregulates T-box 3, a known transcriptional repressor that interacts with the pluripotency factors NANOG and OCT4 to promote the maintenance of pluripotency [21, 22]. Moreover, the AKT/GSK3β pathway is involved in β-catenin phosphorylation and regulates β-catenin to affect ubiquitin-mediated protein degradation. Accumulation of β-catenin by inhibition of GSK3β activity promotes the translocation of β-catenin into the nucleus [23]. Nuclear β-catenin then interacts with TFs and co-activators to promote Wnt target gene expression [24]. Simultaneously, nuclear β-catenin protects against apoptosis by deletion of p53 and p21, thereby increasing reprogramming efficiency [25].

Hair follicles are an easily accessible rich source of autologous stem cells, exhibiting tremendous advantages over other cell sources in various clinical applications. Indeed, the use of hair follicle mesenchymal stem cells (HF-MSCs) as a cell source for skin wound healing, hair follicle regeneration, nerve repair, cardiovascular tissue engineering, and gene therapy has shown remarkable success [26–29]. In a previous study, we successfully use transgenic HF-MSCs overexpressing the release-controlled insulin gene to reverse hyperglycemia and decrease mortality rates in streptozotocin-induced diabetic mice [30]. However, the limited differentiation potential of HF-MSCs restricts their potential applications. Therefore, we reprogrammed HF-MSCs to generate iPSCs that were indistinguishable from hESCs in terms of colony morphology and expression of specific hESC surface markers by lentiviral transduction with SOMK, and these HF-iPSCs could be used as alternative cellular tools for inducing hepatocytes in vitro [31, 32]. Maintenance of HF-MSCs self-renewal ability and enhancement of iPSC generation are essential for the applications in stem cell-based regenerative medicine.

In this study, we aimed to further elucidate the applications of HF-MSCs by investigating the roles of PBX1 in regulating the proliferation and reprogramming of human HF-MSCs. Our results provided important insights into the mechanisms mediating the maintenance of HF-MSC self-renewal ability and pluripotency.

## Methods

### Establishment of HF-MSCs

After the approval of the study protocol by the Ethics Committee of Basic College of Medicine, Jilin University, HF-MSC isolation was performed as described previously [30]. Briefly, HFs were rinsed three times in phosphate-buffered saline (PBS) containing 100 IU/mL penicillin and 100 IU/mL streptomycin (Hyclone, Australia), seeded into 24-well plates (Corning, MA, USA) at one hair follicle per well, and cultured in Dulbecco’s modified Eagle’s medium (DMEM)/Ham’s F-12 medium (Life Technologies, USA) containing 10% fetal bovine serum (FBS; Hyclone, USA) and 4 ng/mL basic fibroblast growth factor (bFGF; Invitrogen, USA) at 37 °C in an incubator with an atmosphere containing 5% CO_2_. When HF-MSCs proliferated to 80% confluence, they were subcultured. HF-MSCs were used for experiments at passages 3–8.

### Immunofluorescence staining and flow cytometry

For immunofluorescence staining, HF-MSCs or HF-iPSCs were fixed with 4% paraformaldehyde for 15 min at room temperature, blocked with 1% bovine serum albumin (Roche Diagnostics, France), and incubated with primary antibodies against CD90, CD105, CD31 (Bioscience, CA, USA), CD44 (R&D Systems, UK), CD73 (Life Technologies, USA), stage-specific embryonic antigen (SSEA) 3, SSEA4 (Developmental Studies Hybridoma Bank, USA), TRA-1-60, TRA-1-81 (Chemicon, USA), NANOG (R&D Systems), and OCT4 (Santa Cruz Biotechnology, Santa Cruz, CA, USA) at 4 °C overnight. The next day, Alexa Fluor 488-conjugated goat anti-mouse/rabbit antibodies were used to detect the primary antibodies (Cell Signaling Technology, Danvers, MA, USA). HF-MSCs were then counterstained with DAPI (Life Technologies, USA) and imaged using fluorescence microscopy (Olympus, Japan). For flow cytometry, HF-MSCs were collected by centrifugation, fixed with paraformaldehyde, blocked with bovine serum albumin, and incubated with primary and secondary antibodies as described above. HF-MSCs were then subjected to flow cytometry (FACS Calibur flow cytometer; BD Biosciences, San Jose, CA, USA) and analyzed using FlowJo software.

### Analysis of the multipotency of HF-MSCs

For adipogenic differentiation assays [30], HF-MSCs were cultured in adipogenic differentiation medium consisting of high-glucose DMEM (Life Technologies) containing 10% FBS (Hyclone), 1 mM dexamethasone, 0.5 mM isobutylmethylxanthine, 10 mM insulin, and 200 mM indomethacin (Sigma-Aldrich, MO, USA). Two weeks after adipogenic induction, Oil red O (Sigma-Aldrich) staining was performed to inspect intracellular lipid droplets.

For osteogenic differentiation assays, HF-MSCs were cultured in high-glucose DMEM containing 10% FBS, 0.1 mM dexamethasone, 50 mM ascorbate-2-phosphate, and 10 nM β-glycerophosphate (Sigma-Aldrich) for 4 weeks. At the end of culture, Alizarin red S (Sigma-Aldrich) staining was performed to inspect the formation of calcium nodules.

### Cell proliferation assay

A Cell Counting Kit-8 (CCK-8; Dojindo, Japan) was used to detect the proliferation of HF-MSCs. Briefly, 2 × 10^3^ cells were seeded in 96-well plates in triplicate and cultured in DMEM/F-12 medium supplemented with 4 ng/mL bFGF and 10% FBS. After 24, 48, 72, and 96 h, CCK-8 reagent was added to each well, and plates were incubated for an additional 2 h. At the end of incubation, the absorbance of the supernatant from each well was measured using a microplate reader (Synergy H1; Biotek, USA) at 450 nm. The results were plotted as the means ± standard deviations from three separate experiments.

### Cell cycle assay

HF-MSCs were transduced with lentiviruses encoding *PBX1*, *PBX1* short hairpin RNA (shRNA), or empty vector. After cells proliferated to 80% confluence, they were collected (1 × 10^6^) by trypsin digestion and centrifugation, washed with cold PBS, and fixed with 70% ice-cold ethanol for 1 h at 4 °C. Finally, the cells were washed with PBS three times and incubated in 500 μL propidium iodide solution containing RNase (BD, USA) for 15 min at room temperature. After incubation, HF-MSCs were washed with PBS and subjected to flow cytometry. ModFitLT was used to estimate G_0_/G_1_/S/G_2_/M phases of the cell cycle. The cell proliferation index (PI) was calculated as follows: PI = (S + G_2_/M)/(G_0_/G_1_ + S + G_2_/M) × 100%.

### Lentiviral vector construction and HF-iPSC generation

The lentiviral vector pLV-CMV-CDNA-IRES-EGFP encoding *PBX1* was obtained from Youbio (China), and the vector pLV-EF1α-CDNA-IRES-EGFP encoding one of the four transcription factors (*OCT4*, *SOX2*, *c-MYC*, or *KLF4*) was obtained from the Xiaolei Group (Shanghai Institute of Biochemistry and Cell Biology, Chinese Academy of Sciences, Shanghai, China). *PBX1* and *NANOG* shRNA sequences were cloned into the lentiviral vector GV115 (GNEN, China). The sequences targeted by shRNA were as follows: *PBX1* (GATCCTGCGTTCCCGATTT) and *NANOG* (TAAACTACTCCATGAACAT). For the preparation of the lentivirus, 10 μg lentiviral vector was cotransfected with 7.5 μg pMD2.G and 2.5 μg psPAX2 (Addgene) into human embryonic kidney 293T cells (obtained from the Xiaolei Group) in T75 flasks using Lipofectamine 3000 transfection reagent (Invitrogen). After the measurement of each lentiviral titers, HF-MSCs were transduced with a cocktail of lentivirus carrying *SOX2*, *OCT4*, *c-MYC*, and *KLF4* (SOMK) or *SOX2*, *OCT4*, *c-MYC*, *KLF4*, and *PBX1* (SOMKP). Forty-eight hours post-transduction, 5 × 10^4^ cells HF-MSCs were plated in a 60-mm dishes (Nest, China). The next day, the medium was aspirated and replaced with hESC culture medium (80% DMEM/F-12 supplemented with 20% knockout serum replacement, 1% nonessential amino acids, 1 mM l-glutamine, 4 ng/mL human bFGF, and 0.1 mM β-mercaptoethanol (Invitrogen). HF-MSCs transduced with SOMK, SOMKP, or SOMK-*PBX1* shRNA were cultured in hESC culture medium for 32 days, and colonies showing alkaline phosphatase staining were designated as HF-iPSCs.

### Teratoma formation and karyotype assays

HF-iPSCs were subcutaneously injected into non-obese diabetic/severe combined immune-deficient (NOD/SCID) mice (HFK, China) at 5 × 10^6^ HF-iPSCs/mouse. Teratomas developed in NOD/SCID mice at 8 weeks after HF-iPSC injection. Teratomas were then harvested and processed for hematoxylin-eosin (H&E) staining and karyotyping. For H&E staining, teratomas were fixed in 10% formalin, embedded in paraffin, sectioned at 5 μm thickness, stained with H&E, and imaged using microscopy (Olympus). Karyotyping was performed at the Department of Genetics, College of Basic Medical Sciences, Jilin University, using standard protocols for high-resolution G-banding.

### Dual luciferase reporter assay

HF-MSCs transduced with lentiviruses encoding a cocktail of transcription factors (SOMK, SOMKP, or SOMK-*PBX1* shRNA) were seeded in 6-well plates and cultured in the hESC culture medium. Twenty-four hours later, 500 ng pNanog-Luc plasmid (Plasmid 25900; Addgene) and 50 ng pRL-TK plasmid (Youbio) were cotransfected into HF-MSCs in triplicate using Lipofectamine 3000. At 24 h after transfection, HF-MSCs were lysed, and dual firefly/Renilla luciferase reporter assays were performed (Beyotime, China) according to the manufacturer’s instructions using a microplate reader (Synergy H1; Biotek). Relative luciferase units were calculated as the ratio of firefly to Renilla luciferases after normalization to the control group (SOMK).

### Apoptosis assays

Apoptosis analysis was performed using an Annexin V-APC/7-AAD Apoptosis Detection Kit (Sungene, China) according to the manufacturer’s instructions. Briefly, 1 × 10^5^ HF-MSCs transduced with lentiviruses encoding a cocktail of TFs were suspended in 100 μL binding buffer containing 5 μL Annexin V-APC and incubated for 10 min in the dark at room temperature. After incubation, 5 μL 7-AAD was added, and HF-MSCs were incubated for an additional 5 min at room temperature. Cells were then subjected to flow cytometry (BD Biosciences).

### Caspase 3 activity detection

To evaluate the activity of caspase 3, HF-MSCs were collected on days 7 and 21 after transduction with lentiviruses encoding a cocktail of TFs and then washed with PBS. Next, 5 × 10^5^ HF-MSCs were lysed with 60 μL lysis buffer and centrifuged. Forty microliters of HF-MSCs lysate was then added to 50 μL reaction buffer, and 10 μL Ac-DEVD-pNA (Beyotime) was added to the mixture. Lysates were incubated at 37 °C for 2 h. The colorimetric reaction was measured at 405 nm in a microtiter plate reader.

### Quantitative polymerase chain reaction and western blotting

Total RNA was extracted from HF-iPSCs and hESCs-X01 (obtained from the Xiaolei Group) using TRIzol reagent (Invitrogen), reverse transcribed into cDNA, and subsequently used as a template PCR (TransGen Biotech, China). qPCR was performed with a kit (Roche, CH), according to the manufacturer’s instructions, using a 7300 Real-Time PCR System (ABI, USA). Data were analyzed by the comparative threshold cycle (Ct) method, and the relative expression was calculated as 2^−ΔCt^, with glyceraldehyde 3-phosphate dehydrogenase (*GAPDH*) as an endogenous control. The primers used for qPCR are listed in Table 1.

**Table 1:** Primers for qPCR

Gene	Forward primers (5′ to 3′)	Revers primers(5′ to 3′)
PBX1	GAGACGGAATTTCAACAAGCA	GTTTGATACCTGGGAGACTG
Endo-OCT4	GGGAGGAGCTAGGGAAAGAAAACCT	GAACTTCACCTTCCCTCCAACCAGT
Endo-SOX2	TTAGAGCTAGTCTCCAAGCGACGA	CCACAGAGATGGTTCGCCAG
NANOG	ATGGAGGGTGGAGTATGGTTGG	AGGCTGAGGCAGGAGAATGG
CRIPTO	TACCTGGCCTTCAGAGATGACA	CCAGCATTTACACAGGGAACAC
FOXD3	AAGCCCAAGAACAGCCTAGTGA	GGGTCCAGGGTCCAGTAGTTG
LIN28	CAGGTGCTACAACTGTGGAGG	GCACCCTATTCCCACTTTCTCC
FGF4	CTACAACGCCTACGAGTCCTACA	GTTGCACCAGAAAAGTCAGAGTTG
ESG1	ATATCCCGCCGTGGGTGAAAGTTC	ACTCAGCCATGGACTGGAGCATCC
GAPDH	CCATGTTCGTCATGGGTGTGA	CATGGACTGTGGTCATGAGT

For western blotting, 1.5 × 10^6^ cells were lysed in 250 μL RIPA (Beyotime Biotechnology, China) supplemented with 1% protease inhibitor cocktail (CoWin Biosciences, China) and 1% phosphatase inhibitor cocktail (CoWin Biosciences, China) on ice for 20 min and centrifuged at 15,000*g* for 20 min at 4 °C. The extraction of nucleoprotein was performed with a kit (Beyotime Biotechnology), according to the manufacturer’s instructions. Forty micrograms of protein was separated using precast gels (Biofuraw, China) and electrotransferred to polyvinylidene fluoride membranes (Millipore, The Netherlands). The membranes were incubated with primary antibodies targeting PBX1, AKT, phospho-AKT (Ser473), GSK-3β, phospho-GSK-3β (Ser9), β-catenin, p21, caspase-3, poly (ADP ribose) polymerase 1 (PARP1), cyclin D1 (Cell Signaling Technology; 1:1000 dilution), p53 (Santa Cruz Biotechnology; 1:1000 dilution), p16, NANOG (ProteinTech, USA; 1:2000 dilution), BAX, BCL2 (Abcam, UK; 1:1000 dilution), HISTONE, and GAPDH (ProteinTech; 1:4000 dilution). The membranes were incubated with enhanced chemiluminescence reagent (TransGen Biotech), and proteins were visualized using a Tanon 5200 instrument (Tanon, China). The grayscale intensities of the results were analyzed using Tanon Gis analytical software.

### Statistical analysis

Results are presented as means ± standard deviations. All data are from at least three independent experiments. Comparisons between the two groups were performed with independent sample *t* tests, and differences among multiple groups were compared with one-way analysis of variance. Differences with *P* values of less than 0.05 were considered statistically significant.

## Results

### HF-MSCs displayed surface marker of MSCs and exhibited adipogenic and chondrogenic differentiation potential

**Fig. 1:**
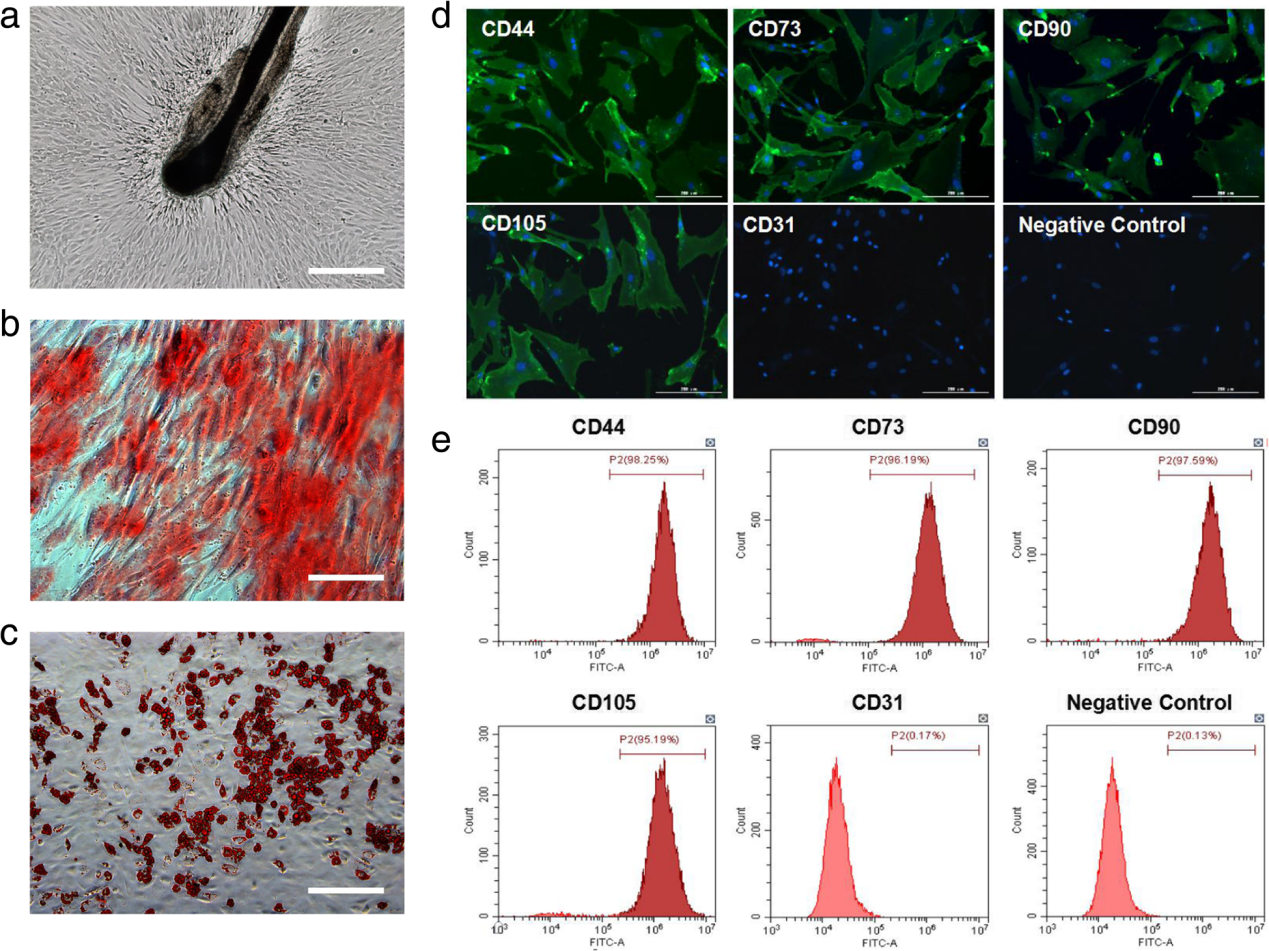
Isolation and characterization of human hair follicle mesenchymal stem cells (HF-MSCs). **a** HF-MSCs, resembling typical fibroblast-like cells, spread out from the hair follicle (bar, 200 μm). The multipotent differentiation potential of HF-MSCs was determined. **b** After 4 weeks of induction, calcium nodules were demonstrated by Alizarin red staining (bar, 100 μm). **c** After 3 weeks of induction, the number of intracellular lipid droplets was detected by Oil red O staining (bar, 200 μm). **d**, **e** Immunofluorescence and flow cytometric analysis of cell surface markers on HF-MSCs

Ten days after the initiation of HF culture, fibroblast-like cells migrated outwards from HFs (Fig. 1a). Immunofluorescence staining combined with flow cytometry assays showed that fibroblast-like cells displayed surface markers of MSCs (positive for CD73, CD44, CD90, and CD105 and negative for CD31 (Fig. 1d, e). Under osteogenic culture conditions, the cells changed morphology from that of fibroblast-like to that of osteoblast-like cells and showed high levels of alkaline phosphatase activity (Fig. 1b). Under adipogenic differentiation culture conditions, the cells showed lipid droplet formation in the cytoplasm by Oil red O staining (Fig. 1c). Hair follicle-derived fibroblast-like cells exhibited surface markers of mesenchymal stem cells and display trilineage differentiation potentials toward osteoblasts and adipocytes. Accordingly, these cells were designated as HF-MSCs.

### PBX1 promoted HF-MSC proliferation through activation of the AKT/GSK3β signaling pathway

**Fig. 2:**
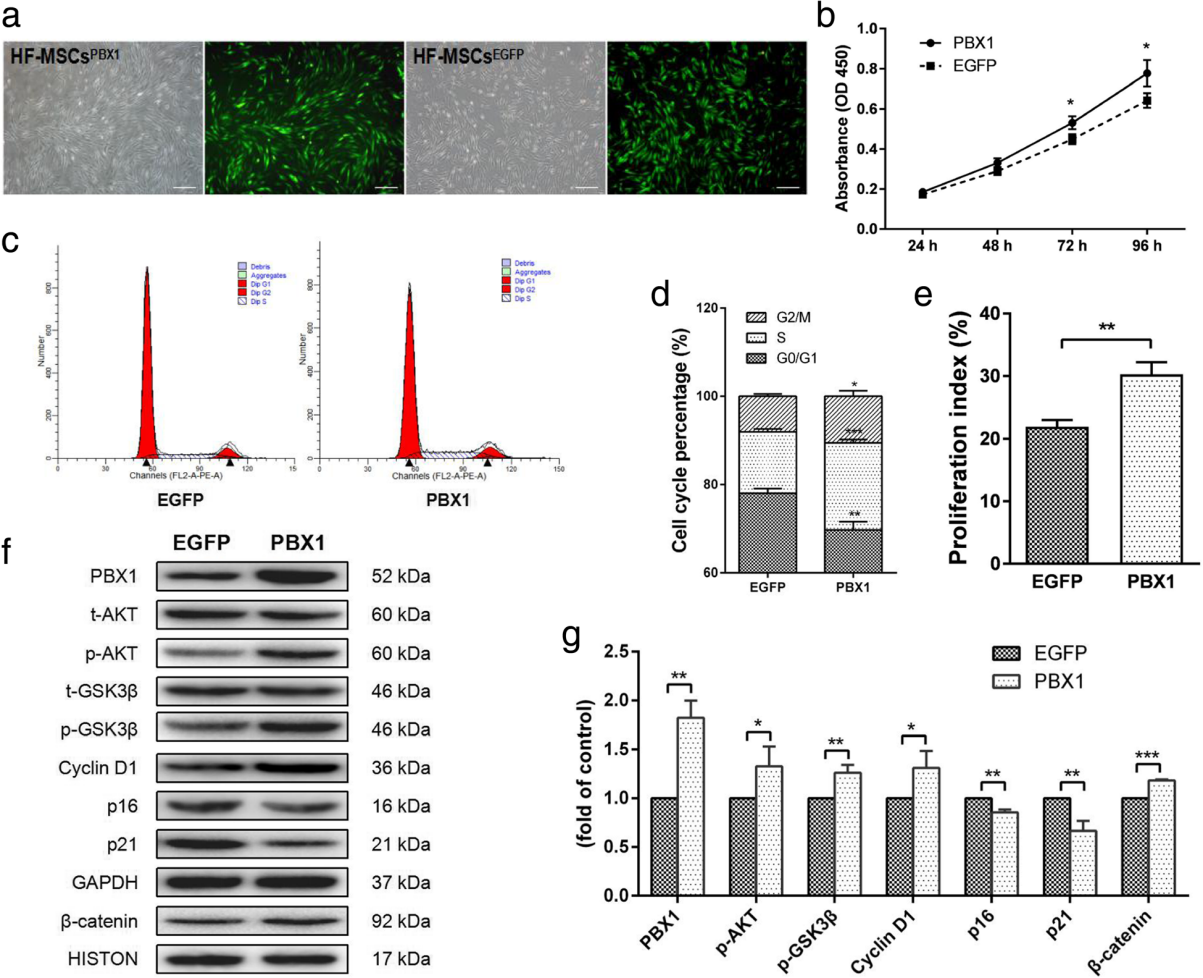
Expression of PBX1 in transduced HF-MSCs increased proliferation capacity. **a** The cell morphologies of PBX1-transduced HF-MSCs (bar, 100 μm). **b** Cell proliferation curve of HF-MSCs^EGFP^ and HF-MSCs^PBX1^. **c** Effects of PBX1 in the cell cycle distribution in HF-MSCs. **d** Percentages of cells in the G_1_, S, and G_2_ phases of the cell cycle and PIs (**e**) of HF-MSCs^EGFP^ and HF-MSCs^PBX1^. **f**, **g** Western blot analysis the levels of phospho-AKT, phospho-GSK3β, cyclin D1, p16, p21, and β-catenin proteins in HF-MSCs^EGFP^ and HF-MSCs^PBX1^. GAPDH and HISTONE were used as endogenous controls for equal loading. The value for HF-MSCs^EGFP^ in the control group transduced by lentiviral vector was set as 1. *P < 0.05; **P < 0.01

To explore the effects of PBX1 on HF-MSC proliferation, HF-MSCs were transduced with a lentiviral vector encoding *PBX1* (HF-MSCs^PBX1^) or empty vector (HF-MSCs^EGFP^; Fig. 2a). Exogenous expression of PBX1 was confirmed by western blotting (Fig. 2f). CCK-8 assays showed that overexpression of PBX1 significantly increased the rate of HF-MSC proliferation at 72 and 96 h after cell seeding (*P* < 0.05, 72 h; *P* < 0.05, 96 h; Fig. 2b). Cell cycle analyses showed that overexpression of PBX1 induced the entry of HF-MSCs from G_0_/G_1_ to S and G_2_/M phases (Fig. 2c, d), with significantly higher PIs (*P* < 0.01; Fig. 2e). Consistent with cell proliferation and cell cycle assays, western blotting showed that PBX1 increased the levels of phospho-AKT (*P* < 0.05), phospho-GSK3β (*P* < 0.01), and cyclin D1 (*P* < 0.05) and promoted β-catenin translocation from the cytoplasm to the nucleus (*P* < 0.001). Moreover, PBX1 expression decreased p16 (*P* < 0.01) and p21 (*P* < 0.01) expression (Fig. 2f, g).

**Fig. 3:**
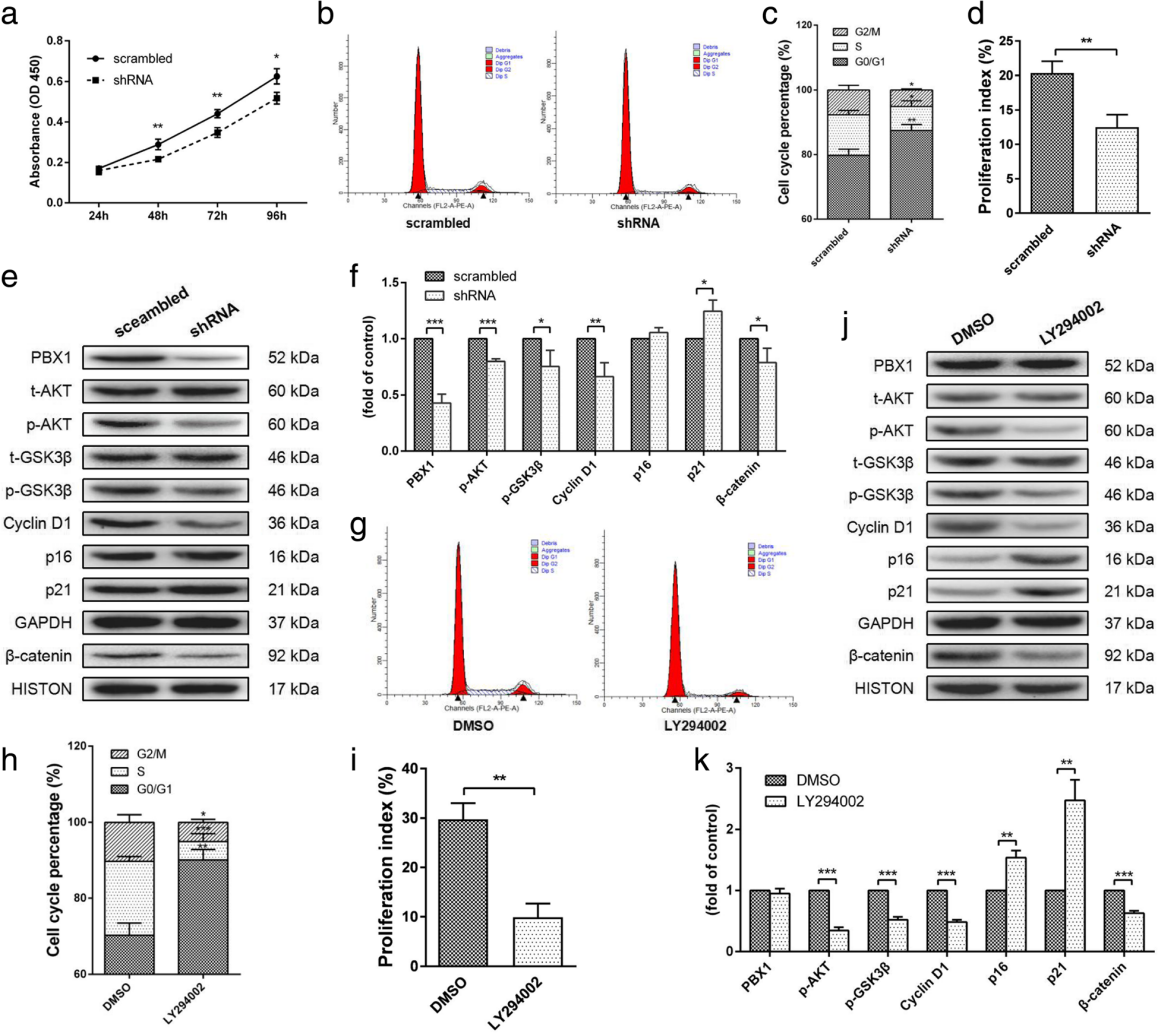
Knockdown of PBX1 in HF-MSCs suppressed proliferation and inhibited the AKT/GSK3β pathway. **a** Cell proliferation curves for HF-MSCs^scrambled^ and HF-MSCs^shRNA^. **b**, **c** Percentages of cells in the G_1_, S, and G_2_ phases of the cell cycle and PIs (**d**) for the HF-MSCs^scrambled^ and HF-MSCs^shRNA^. **e**, **f** Western blot analysis of the levels of phospho-AKT, phospho-GSK3β, cyclin D1, p16, p21, and β-catenin proteins in HF-MSCs^scrambled^ and HF-MSCs^shRNA^. **g**, **h** Percentages of cells in the G_1_, S, and G_2_ phases of the cell cycle and PIs (**i**) for HF-MSCs^PBX1^ cultured with DMSO and LY294002. **j**, **k** Western blot analysis of the levels of phospho-AKT, phospho-GSK3β, cyclin D1, p16, p21, and β-catenin proteins in HF-MSCs^PBX1^ cultured with DMSO and LY294002. *P < 0.05; **P < 0.01; ***P < 0.001

To explore the mechanism through which PBX1 enhanced HF-MSC proliferation, endogenous PBX1 was knocked down by transduction with a lentiviral vector encoding *PBX1* shRNA (HF-MSCs^shRNA^) or scrambled shRNA vector as a control (HF-MSCs^scrambled^). The cell growth rates at 48 h (*P* < 0.01), 72 h (*P* < 0.01), and 96 h (*P* < 0.05) were significantly decreased in HF-MSCs^shRNA^ compared with those in HF-MSCs^scrambled^ (Fig. 3a). Moreover, PBX1 knockdown significantly reduced the percentage of HF-MSCs in the S phase from 12.53% ± 0.782% to 7.39% ± 1.01% (*P* < 0.05) and the PIs from 20.34 ± 0.99 to 12.50 ± 1.05 (*P* < 0.05; Fig. 3b–d). Western blotting showed that PBX1 knockdown resulted in significant decreases in the levels of phospho-AKT, phospho-GSK3β, cyclin D1, and nuclear β-catenin, but increased the expression of the cyclin kinase inhibitor p21 (Fig. 3e, f).

To confirm that the role of PBX1 in enhancing HF-MSC proliferation involved the activation of the AKT/GSK3β signaling pathway, HF-MSCs^PBX1^ were treated with 10 μM LY294002 for 24 h [18]. Flow cytometry assays showed that LY294002 treatment significantly reduced the percentage of HF-MSCs in the S phase and decreased the PI from 29.77 ± 1.850 to 9.913 ± 1.602 (*P* < 0.005; Fig. 3g–i). Western blotting showed that LY294002 treatment dramatically decreased the levels of cyclin D1 (*P* < 0.001), phospho-AKT (*P* < 0.001), and phospho-GSK3β (*P* < 0.001), but increased the levels of p16 by 1.6-fold (*P* < 0.01) and p21 by 2.5-fold (*P* < 0.01; Fig. 3j, k). However, LY294002 treatment did not cause any significant changes in PBX1 expression, suggesting that PBX1 increased the HF-MSC proliferation through activation of the AKT/GSK3β signaling pathway.

### PBX1 enhanced HF-iPSC generation and upregulated pluripotent gene expression

**Fig. 4:**
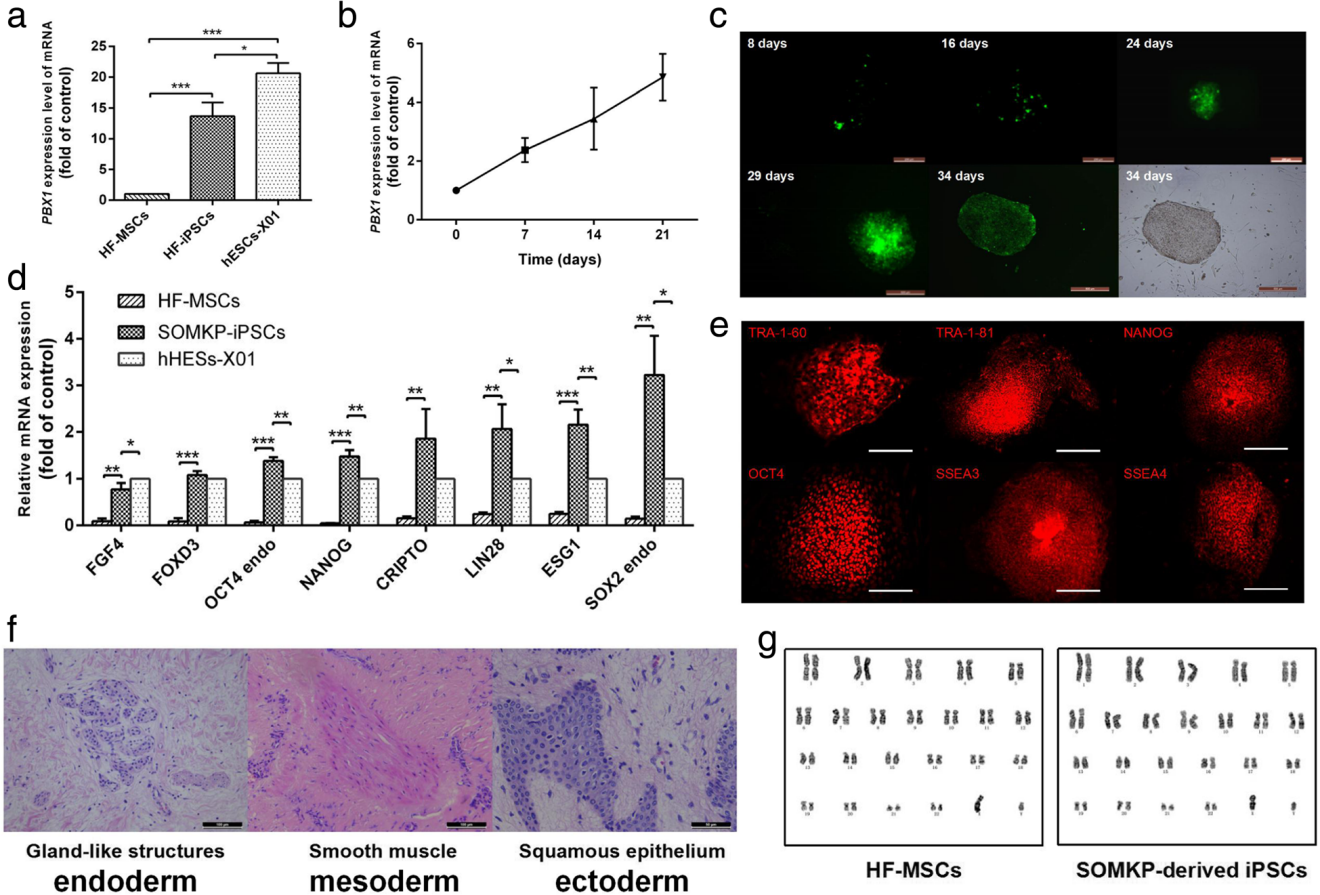
Characterization of PBX1-induced pluripotent stem cells from HF-MSCs. **a** Transcript levels of endogenous *PBX1* in HF-MSCs, HF-iPSCs, and hESCs-X01 were determined by qPCR. The value in HF-MSCs as the control group was set as 1.0. **b** After transduction with SOMK, HF-MSCs were harvested on days 0, 7, 14, and 21, and the expression of endogenous *PBX1* was assessed by qPCR. Data are shown as fold induction compared with that at day 0. **c** The cell morphologies of transduced HF-MSCs were changed by reprogramming at 0 and 34 days after SOMKP transduction (bar, 500 μm). **d** Expression of endogenous pluripotency genes in hESCs and HF-iPSCs^SOMKP^ relative to that in parental somatic cell populations, as determined by qPCR. Data are shown as fold induction compared with that in hESCs-X01. **e** HF-iPSCs^SOMKP^ expressed TRA-1-60, TRA-1-81, SSEA-3, SSEA-4, OCT4, and NANOG, as shown by immunostaining (bar, 200 μm). **f** H&E staining of teratomas obtained from HF-iPSCs^SOMKP^ injected into NOD-SCID mice revealed gland-like structures (endoderm), smooth muscle (mesoderm), and squamous epithelium (ectoderm; bar, 100 μm). **g** Karyotype analysis. HF-MSCs at passage 6 (left) and HF-iPSCs^SOMKP^ at passage 12 (right) showed a normal 46XY karyotype. *P < 0.05; **P < 0.01; ***P < 0.001

**Fig. 5:**
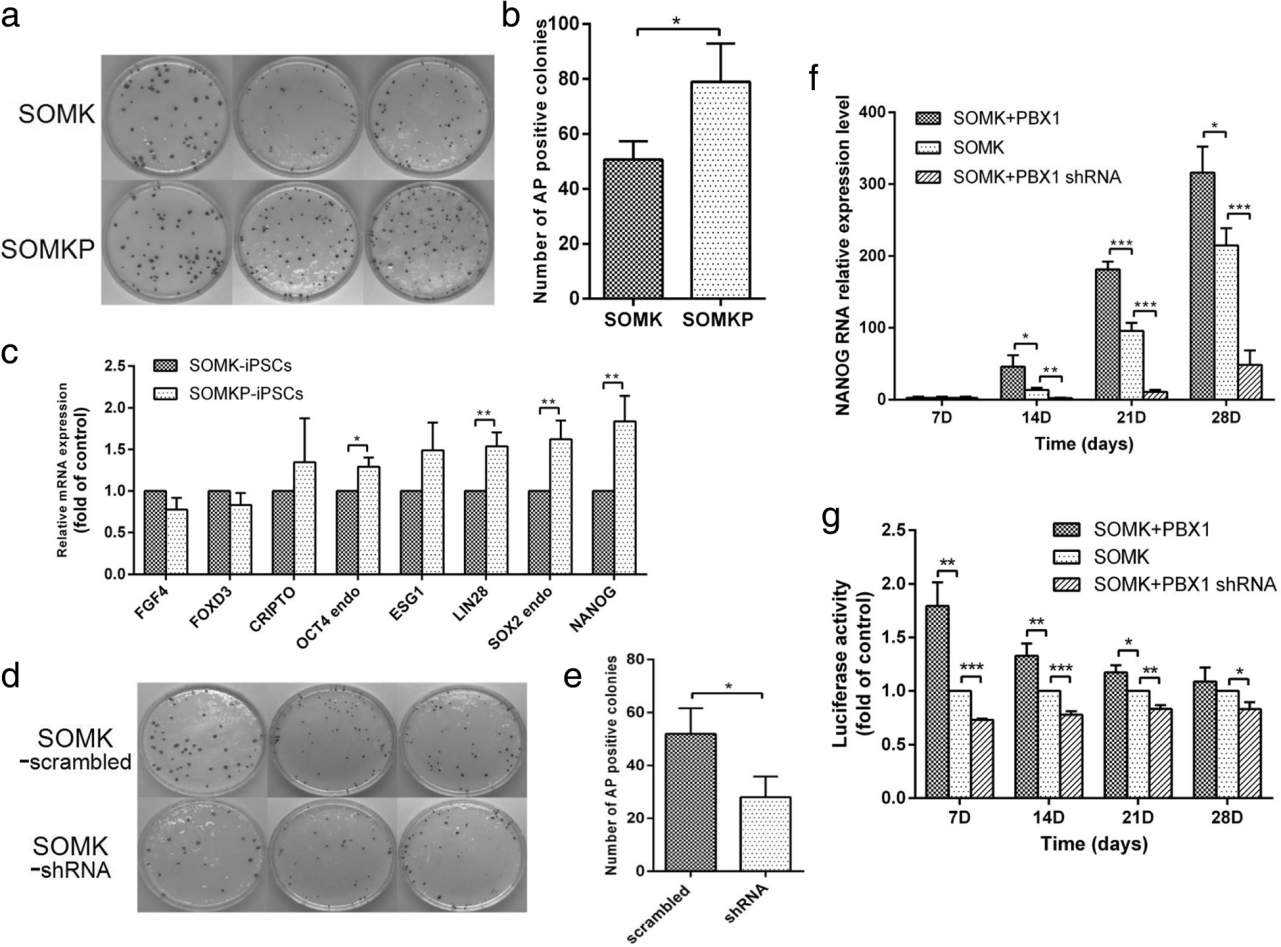
PBX1 enhanced HF-iPSC generation and activated the *NANOG* promoter. **a**, **b** Quantification of the number of alkaline phosphatase-positive colonies 32 days after lentivirus vector-mediated transduction with SOMKP and SOMK vectors into HF-MSCs. **c** Expression of endogenous pluripotency genes in HF-iPSCs^SOMK^ and HF-iPSCs^SOMKP^ was assessed by qPCR. The value in iPSCs^SOMK^ as the control group was set as 1.0. **d**, **e** Quantification of the number of alkaline phosphatase-positive colonies 32 days after lentivirus vector-mediated transduction by SOMK with scrambled shRNA and SOMK with shRNA against *PBX1*. **f** Transduced HF-MSCs were harvested on days 7, 14, 21, and 28 to assess the expression of endogenous *NANOG* by qPCR. Data are shown as fold induction compared with the SOMK control on day 7. **g** Dual-luciferase reporter gene assays were used to assess the activation of the *NANOG* promoter in transduced HF-MSCs on days 7, 14, 21, and 28. The value in SOMK-transduced HF-MSCs as the control group was set as 1.0. *P < 0.05; **P < 0.01; ***P < 0.001

AKT activation enhances the reprogramming of somatic cells into iPSCs [20–22], and our study showed that overexpression of PBX1 activated the AKT/GSK3β signaling pathway, suggesting a role for PBX1 in reprogramming of HF-MSCs into HF-iPSCs. Indeed, the qPCR analysis showed that endogenous *PBX1* levels increased with time during HF-iPSC reprogramming induced by SOMK transduction (Fig. 4b); *PBX1* expression in these cells was significantly higher than that in HF-MSCs (*P* < 0.001) but lower than that in hESCs (Fig. 4a). In addition, compared with HF-MSCs, HF-iPSCs generated by SOMKP transduction (HF-iPSCs^SOMKP^) exhibited high expression of endogenous *FGF4*, *FOXD3*, *NANOG*, *CRIPTO*, *LIN28*, *ESG1*, endo-*OCT4*, and endo*-SOX2* (*P* < 0.05, *P* < 0.001). Additionally, the expression levels of endo-*OCT4*, *NANOG*, *LIN28*, *ESG1*, and endo-*SOX2* were significantly higher than those in hESCs-X01 (*P* < 0.05, *P* < 0.001; Fig. 4d). As expected, HF-iPSCs^SOMKP^ formed typical ESC-like clones, expressing the ESC-related markers SSEA-1, SSEA-4, TRA-1-60, and TRA-1-81, as demonstrated by immunofluorescence staining (Fig. 4c, e). These cells also developed teratomas consisting of ectoderm (squamous epithelium), mesoderm (smooth muscle tissues), and endoderm (gland-like structures) when injected into NOD-SCID mice (Fig. 4f). Moreover, HF-iPSCs^SOMKP^ exhibited a normal male chromosome type (46XY), similar to HF-MSCs, and no chromosomal aberrations were found (Fig. 4g). Interestingly, compared with SOMK transduction, SOMKP transduction significantly increased both HF-iPSC colony formations, from 50.67 ± 3.84 to 79 ± 8.02 (*P* < 0.05; Fig. 5a, b), and increased the expression levels of the endogenous *OCT4*, *LIN28*, *SOX2*, and *NANOG* genes (*P* < 0.05, *P* < 0.01; Fig. 5e). In contrast, knockdown of PBX1 with *PBX1* shRNA significantly decreased SOMK-induced HF-iPSC colony formation, from 52 ± 5.5 to 28 ± 4.5 (*P* < 0.05; Fig. 5d, e).

NANOG is a core TF involved in the maintenance of the pluripotent state in hESCs and reprogramming of somatic cells into iPSCs. Thus, we next evaluated the expression of the *NANOG* by qPCR. The results showed that *NANOG* expression in HF-iPSCs induced by either SOMK or SOMKP transduction increased over time from day 14 to day 28. SOMKP transduction significantly increased *NANOG* expression on days 14 (*P* < 0.05), 21 (*P* < 0.001), and 28 (*P* < 0.05) compared with SOMK transduction. However, knockdown of PBX1 with *Pbx1* shRNA significantly decreased *NANOG* expression during reprogramming (*P* < 0.01, *P* < 0.001; Fig. 5f). Dual-luciferase assays showed that compared with SOMK, SOMKP transduction significantly increased *NANOG* promoter activities by 1.74-, 1.46-, and 1.25-fold during reprogramming of HF-MSCs into HF-iPSCs on days 7 (*P* < 0.01), 14 (*P* < 0.01), and 21 (*P* < 0.05), whereas knockdown of PBX1 significantly decreased *NANOG* promoter activities on days 7 (*P* < 0.001), 14 (*P* < 0.001), 21 (*P* < 0.01), and 28 (*P* < 0.05; Fig. 5g). These findings suggested a role for PBX1 in the activation of the pluripotency-related gene *NANOG* during iPSC reprogramming.

### PBX1 enhanced HF-iPSC generation through activation of the AKT/GSK-3β signaling pathway

**Fig. 6:**
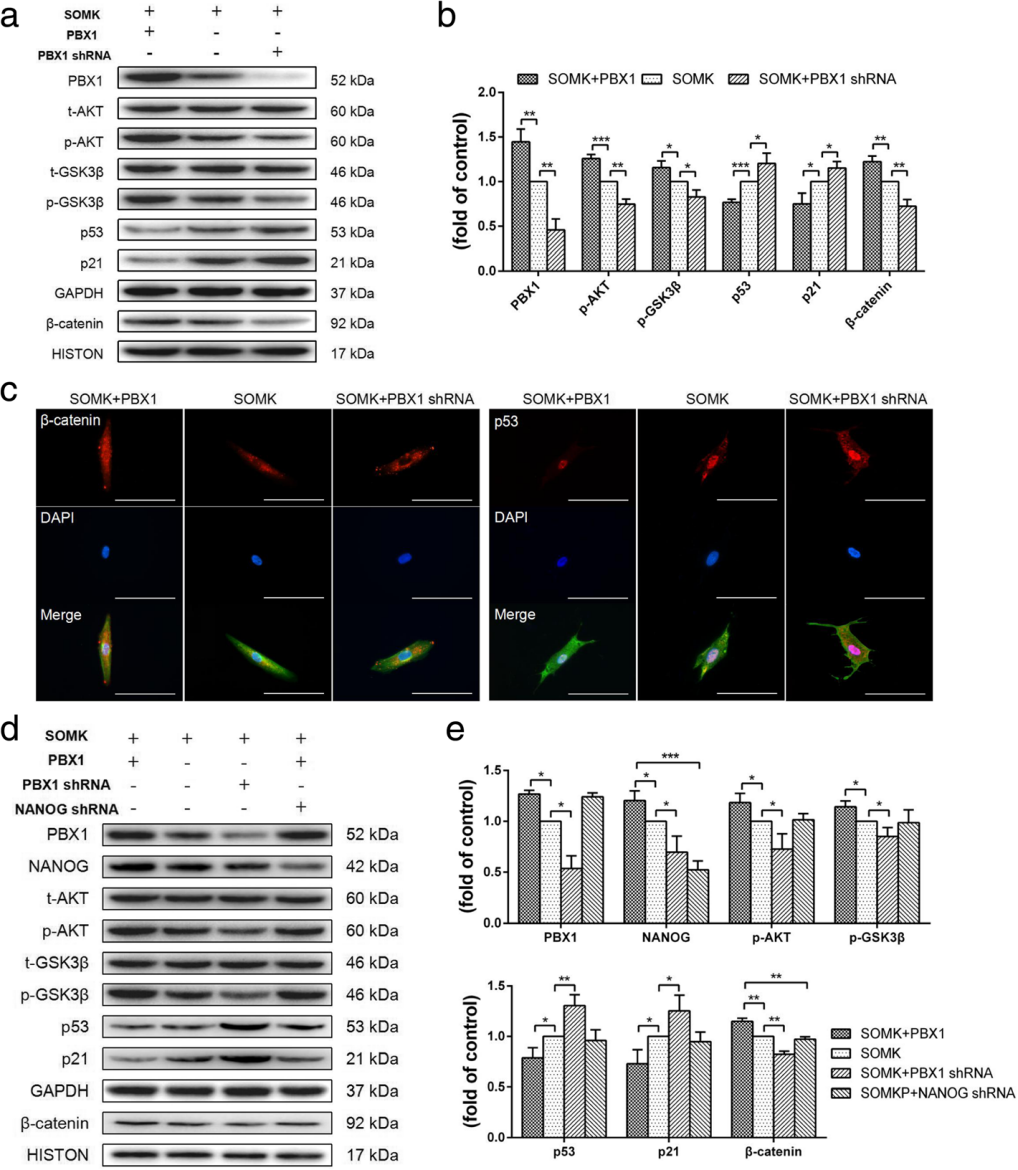
PBX1 activated the ATK/GSK3β signaling pathway in induced pluripotent stem cells. **a**, **b** Transduced HF-MSCs were harvested on day 7 to assess the levels of phospho-AKT, phospho-GSK3β, p16, p21, and β-catenin. GAPDH and HISTONE were used as loading controls. The value in SOMK-transduced HF-MSCs as the control group was set as 1.0. **c** Immunofluorescence analysis of β-catenin and p53 expression and localization in transduced HF-MSCs (bar, 100 μm). **d**, **e** Transduced HF-MSCs were harvested on day 21 to assess the levels of phospho-AKT, phospho-GSK3β, p16, p21, NANOG, and β-catenin. GAPDH and HISTONE were used as endogenous controls for equal loading. The value in SOMK-transduced HF-MSCs as the control group was set as 1.0. *P < 0.05; **P < 0.01; ***P < 0.001

To dissect the mechanisms through which PBX1 enhanced HF-iPSC generation, the relationship between PBX1 and the AKT/GSK3β signaling pathway was explored during HF-iPSC reprogramming. Western blotting showed that compared with SOMK, SOMKP transduction significantly increased the phosphorylation of AKT and GSK3β, promoted the nuclear translocation of β-catenin, and downregulated the p53 and p21 expression during reprogramming on days 7 and 21 (Fig. 6a–e). Inhibition of endogenous PBX1 expression decreased the phosphorylation of AKT and GSK3β, but upregulated the p53 and p21 expression and decreased the nuclear translocation of β-catenin. Immunofluorescence staining showed that PBX1 promoted the accumulation of β-catenin in the cytoplasm and nucleus. In contrast, knockdown of PBX1 inhibited the accumulation of β-catenin in the cytoplasm and nucleus but promoted the accumulation of p53 in the cytoplasm and nucleus. Surprisingly, we found that NANOG expression was positively correlated with PBX1 expression during reprogramming on day 21 and that knockdown of NANOG with *NANOG* shRNA did not cause any significant changes in PBX1, phospho-AKT, or phospho-GSK3β levels. However, a significant decrease in β-catenin nuclear translocation was observed (*P* < 0.01; Fig. 6d, e).

**Fig. 7:**
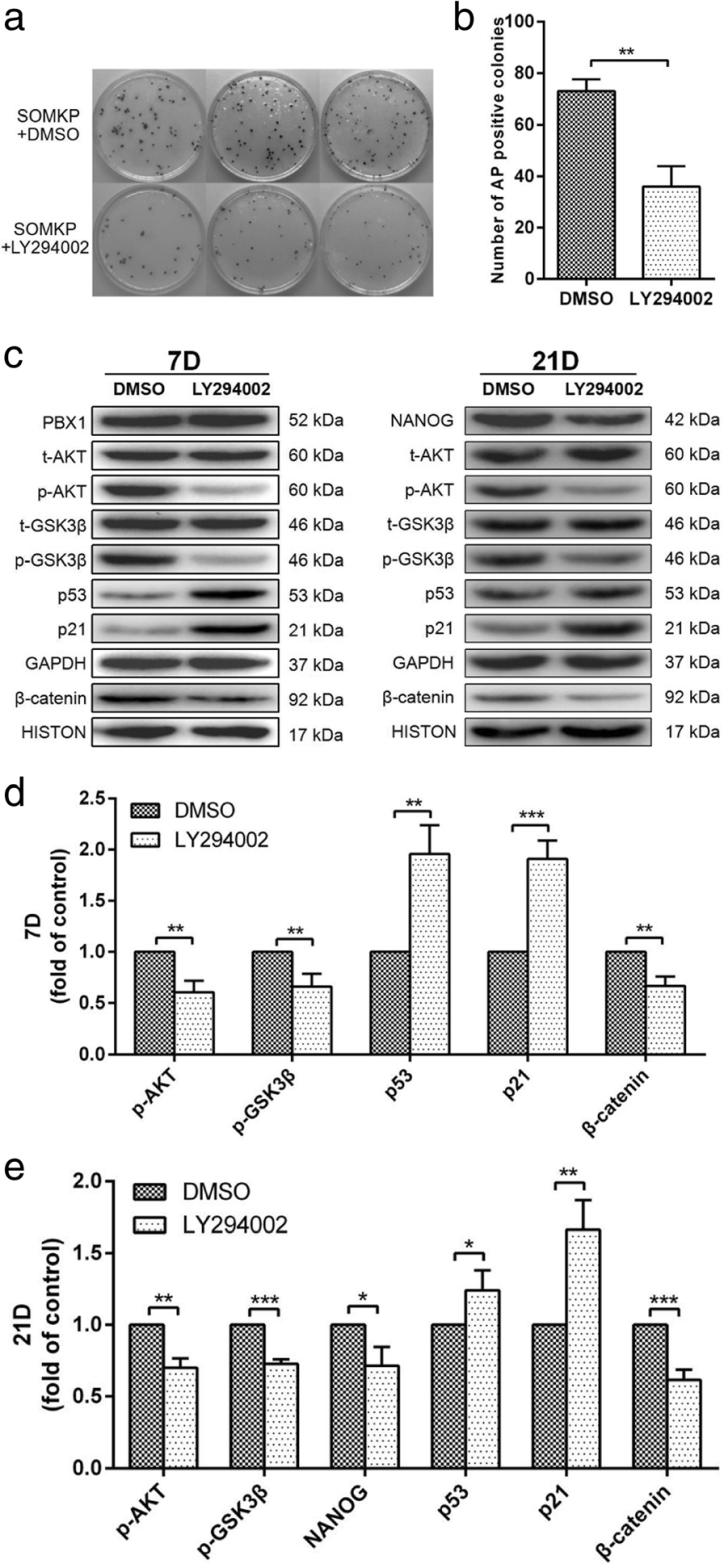
Inhibition of the AKT/GSK3β signaling pathway activated by the overexpression of PBX1 reduced the generation of iPSCs. **a**, **b** Quantification of the number of alkaline phosphatase-positive colonies 32 days after lentivirus vector-mediated transduction by SOMKP with DMSO and LY294002. **c** SOMKP transduced HF-MSCs cultured with LY294002 were harvested on days 7 (**d**) and 21 (**e**) to assess the levels of phospho-AKT, phospho-GSK3β, p16, p21, NANOG, and β-catenin. GAPDH and histone were used as endogenous controls for equal loading. The value in SOMKP-transduced HF-MSCs cultured with DMSO as the control group was set as 1.0. *P < 0.05; **P < 0.01; ***P < 0.001

In order to determine whether PBX1 enhanced HF-iPSC generation via activation of the AKT/GSK3β pathway, the AKT signaling pathway was blocked using the PI3K/AKT inhibitor LY294002 during HF-iPSC reprogramming induced by SOMKP. As expected, LY294002 treatment significantly decreased the numbers of HF-iPSC colony from 73 ± 2.64 to 36 ± 4.583 (*P* < 0.01; Fig. 7a, b). Western blotting showed that LY294002 treatment not only reduced phospho-AKT levels but also decreased NANOG and phopsho-GSK3β levels and blocked β-catenin nuclear translocation. In contrast, LY294002 treatment significantly increased the expression of p53 and p21 during reprogramming on days 7 and 21 (Fig. 7c–e). These results suggested that the AKT/GSK3β pathway acted downstream of PBX1 to regulate NANOG expression and cell reprogramming.

### PBX1 reduced apoptosis during HF-iPSC reprogramming

**Fig. 8:**
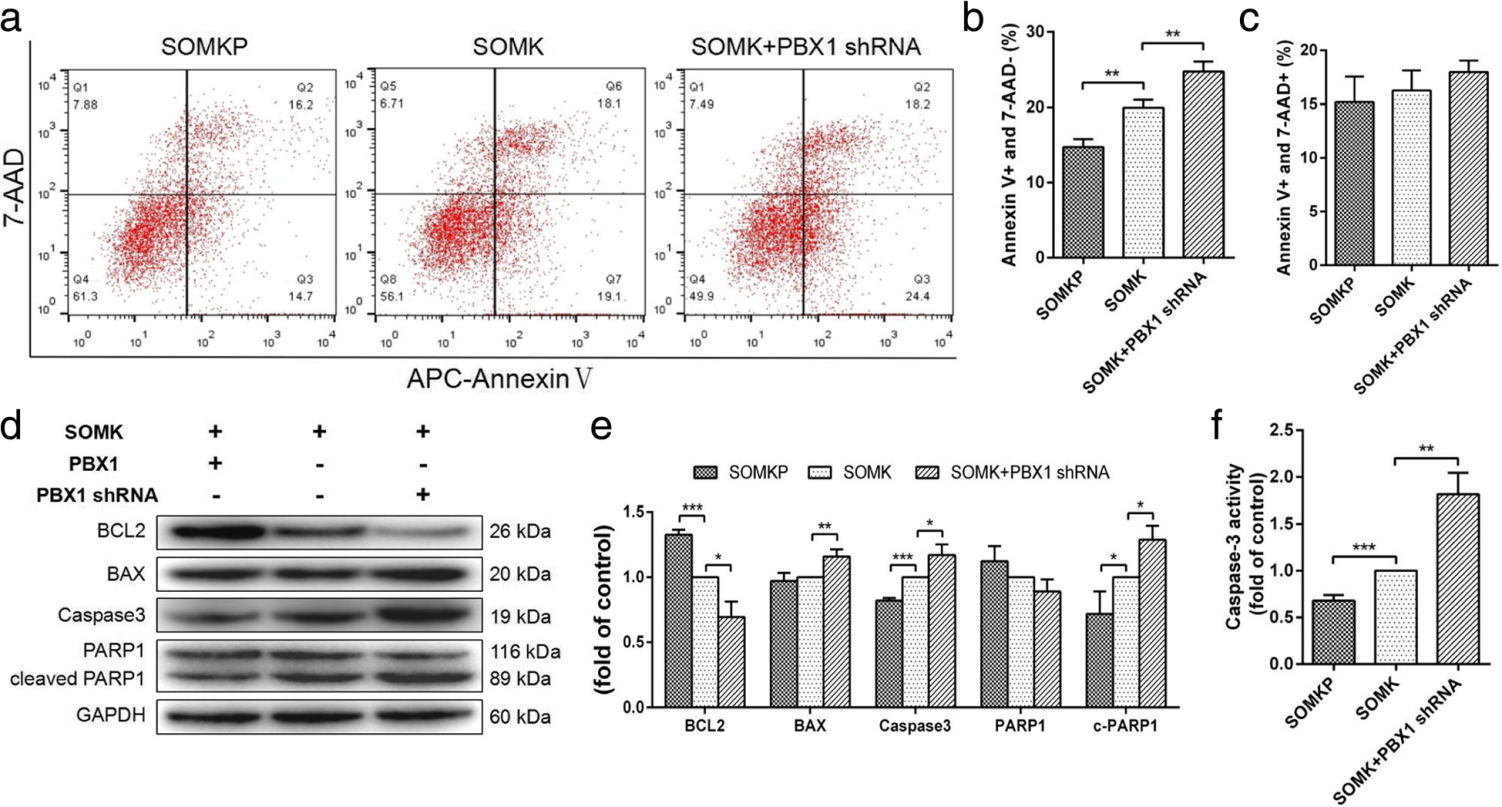
PBX1 reduced apoptosis during the early stage of reprogramming. **a** Apoptosis of transduced HF-MSCs on reprogramming day 7. **b** Quantitative analysis of the proportion of early apoptotic cells (APC Annexin V^+^ and 7-AAD^−^). **c** Quantitative analysis of the proportion of late apoptotic cells (APC Annexin V^+^ and 7-AAD^+^). **d**, **e** Western blotting was used to detect the expression of apoptosis-related proteins (BCL-2, BAX, Caspase3, and PARP1) in transduced HF-MSCs on reprogramming day 7. **f** Caspase3 activity in transduced HF-MSCs on reprogramming day 7 is shown as the fold change compared with the control. The value in SOMK-transduced HF-MSCs as the control group was set as 1.0. *P < 0.05; **P < 0.01; ***P < 0.001

**Fig. 9:**
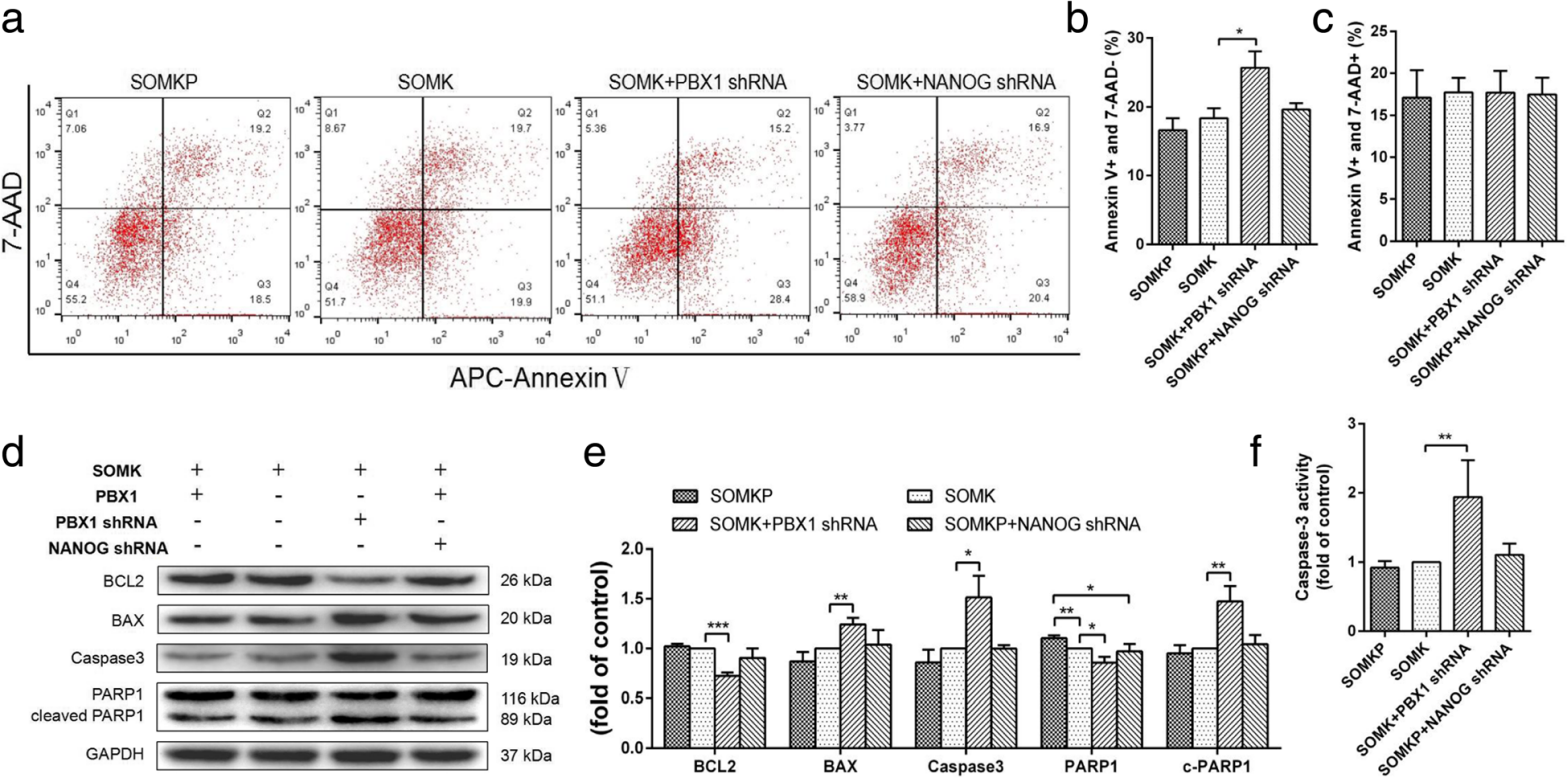
Knockdown of PBX1 promoted apoptosis during reprogramming. **a** Apoptosis in transduced HF-MSCs during reprogramming on day 21. **b** Quantitative analysis of the proportion of early apoptotic cells (APC Annexin V^+^ and 7-AAD^−^). **c** Quantitative analysis of the proportion of late apoptotic cells (APC Annexin V^+^ and 7-AAD^+^). **d**, **e** Western blotting analysis of the expression levels of apoptosis-related proteins (BCL-2, BAX, caspase3, and PARP1) in transduced HF-MSCs during reprogramming on day 21. **f** Caspase3 activity in transduced HF-MSCs during reprogramming on day 21, shown as the fold change compared with the control. The value for SOMK-transduced HF-MSCs as the control group was set as 1.0. *P < 0.05; **P < 0.01; ***P < 0.001

Apoptosis is a key resistance mechanism in somatic cell reprogramming [33, 34]. Therefore, the role of PBX1 in the regulation of apoptosis during HF-iPSC reprogramming was explored. Flow cytometry showed that compared with SOMK, SOMKP transduction significantly reduced the percentage of AnnexinV^+^/7-AAD^−^ cells from 19.97% ± 0.6% to 14.73% ± 0.61% during reprogramming on day 7 (*P* < 0.01); however, no significant differences were detected on day 21 (Fig. 8a–c). Additionally, SOMKP transduction did not significantly alter the percentages of AnnexinV^+^/7-AAD^+^ cells during reprogramming on days 7 and 21. To confirm the role of PBX1 in reducing HF-MSC apoptosis during HF-iPSC reprogramming, PBX1 was knocked down with *PBX1* shRNA. Flow cytometry showed that PBX1 knockdown increased the percentage of AnnexinV^+^/7-AAD^−^ cells from 19.97% ± 0.6% to 24.73% ± 0.77% (*P* < 0.01) during reprogramming on day 7 and from 18.37% ± 0.84% to 25.67% ± 1.386% (*P* < 0.05) during reprogramming on day 21 (Fig. 9a, b). There were no significant differences in the SOMK group with regard to the percentage of AnnexinV^+^/7-AAD^+^ cells (Fig. 9c). Unexpectedly, the knockdown of NANOG did not cause any significant changes in the percentages of AnnexinV^+^/7-AAD^−^ and AnnexinV^+^/7-AAD^+^ cells during reprogramming on day 21. Western blotting showed that compared with SOMK, SOMKP transduction significantly upregulated BCL2 expression, downregulated caspase3 and cleaved PARP1 expression, and decreased caspase3 activity during reprogramming on days 7 (Fig. 8d, e). In contrast, the knockdown of PBX1 downregulated BCL2 expression; upregulated BAX, caspase3, and cleaved PARP1 expression; and increased caspase 3 activity during reprogramming on days 7 and 21 (Fig. 9d, e). Similar to apoptosis assays, the knockdown of Nanog did not cause any significant changes in BCL2, BAX, caspase3, and cleaved PARP1 expression or in caspase 3 activity in HF-MSCs transduced with SOMKP during reprogramming on day 21 (Fig. 9f). Overall, these results demonstrated that overexpression of PBX1 significantly inhibited HF-MSC apoptosis during the early stages of reprogramming and that inhibition of endogenous PBX1 expression promoted apoptosis during reprogramming.

### PBX1 reduced apoptosis by activation of the AKT/GSK3β signaling pathway during HF-iPSC reprogramming

**Fig. 10:**
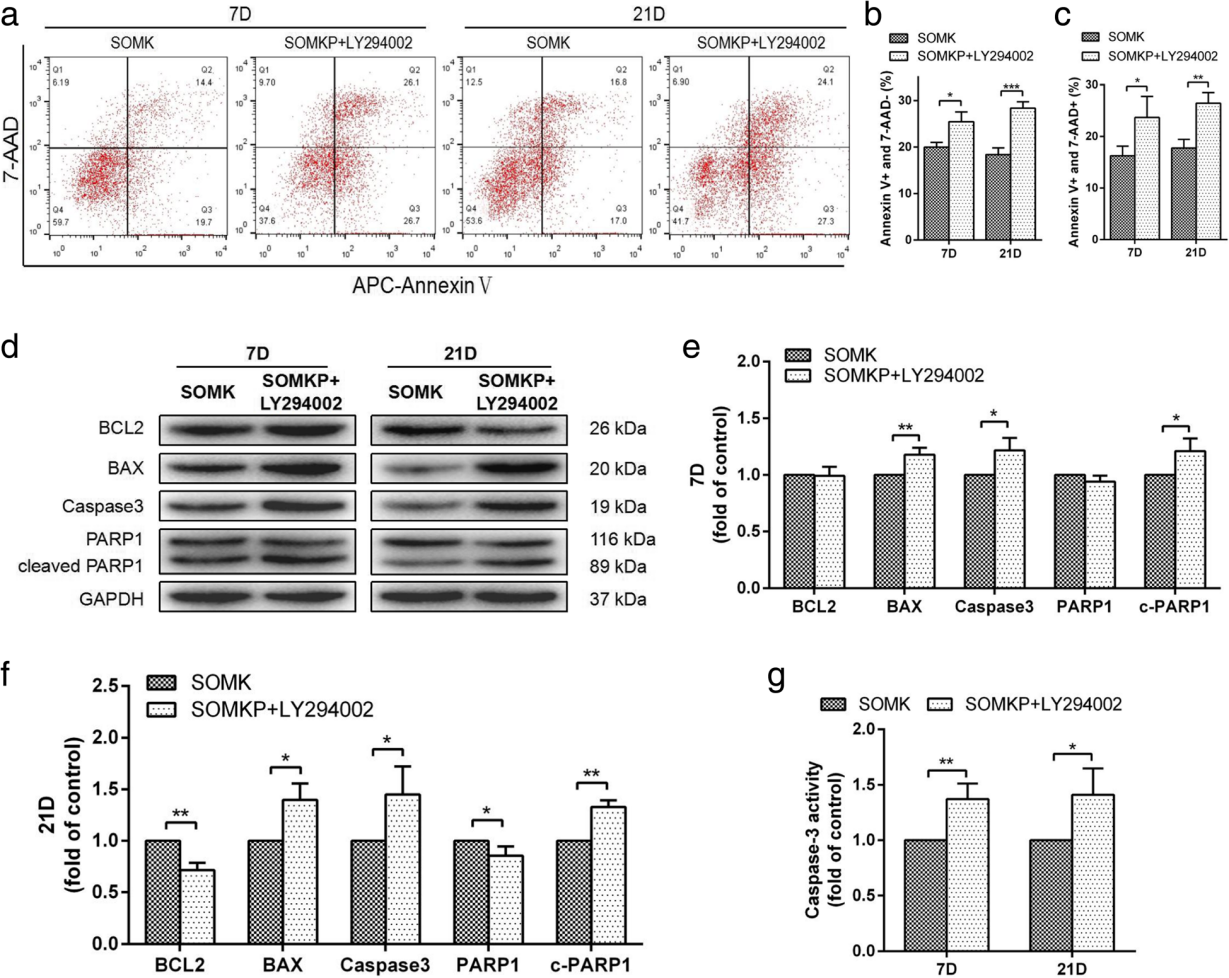
Overexpression of PBX1 inhibited the AKT/GSK3β signaling pathway to promote apoptosis during reprogramming. **a** Apoptosis in SOMK-transduced HF-MSCs and SOMKP-transduced HF-MSCs cultured with LY294002 during reprogramming on days 7 and 21. **b** Quantitative analysis of the proportion of early apoptotic cells (APC Annexin V+ and 7-AAD-). **c** Quantitative analysis of the proportion of late apoptotic cells (APC Annexin V+ and 7-AAD^+^). **d** Western blotting analysis of the expression of apoptosis-related proteins (BCL-2, BAX, caspase3, and PARP1) in transduced HF-MSCs during reprogramming on days 7 (**e**) and 21 (**f**). **g** Caspase3 activity in transduced HF-MSCs during reprogramming on day 21, shown as the fold change compared with the control. The value for SOKM-transduced HF-MSCs as the control group was set as 1.0. *P < 0.05; **P < 0.01; ***P < 0.001

In order to explore whether PBX1 inhibited HF-MSC apoptosis during HF-iPSC reprogramming through the AKT/GSK3β pathway, HF-MSCs were treated with the specific PI3K/AKT inhibitor LY294002 during HF-iPSC reprogramming induced by SOMKP. Flow cytometry revealed that compared with SOMK, LY294002 treatment increased the percentages of both AnnexinV^+^/7-AAD^−^ cells during reprogramming on day 7 (*P* < 0.05) and on day 21 (*P* < 0.001). Additionally, treatment with this inhibitor increased the percentages of AnnexinV^+^/7-AAD^+^ cells during reprogramming on day 7 (*P* < 0.05) and day 21 (*P* < 0.01; Fig. 10a–c). These findings suggested that LY294002 suppress apoptotic protection granted by PBX1 exogenous expression. Moreover, LY294002 treatment significantly increased the caspase3 activity by 1.36-fold (P < 0.01) during reprogramming on day 7 and by 1.4-fold (*P* < 0.05) on day 21 (Fig. 10g). Consistent with flow cytometry assays, western blotting showed that LY294002 treatment significantly increased the expression of the apoptosis-related proteins BAX, caspase3, and cleaved PARP1 during reprogramming on days 7 (Fig. 10e) and 21 (Fig. 10f). These results revealed that the inhibitory activity of the AKT/GSK3β pathway accounted for the effects of overexpression of PBX1 on the protection of HF-MSCs against apoptosis.

## Discussion

HF-MSCs and HF-iPSCs reprogrammed from HF-MSCs offer autologous stem cell sources for tissue repair and regeneration, without induction of immune responses or concerns regarding the ethics of allogeneic implantation. Cell senescence caused by long-term culture significantly reduced the therapeutic potential of stem cells. Moreover, low reprogramming efficiency is a major challenge for the clinical application of HF-iPSCs, thus necessitating the development of novel strategies to enhance HF-MSC proliferation and HF-iPSC generation. TFs are key intrinsic regulators involved in the maintenance of the pluripotent state of stem cells and reprogramming of somatic cells into iPSCs, orchestrating the interaction networks in a temporal and spatial manner to regulate stem cell proliferation and differentiation. In this study, we found that PBX1 promoted cell proliferation and reprogramming in HF-MSCs. Our findings demonstrated that PBX1 enhanced cell cycle progression from G_0_/G_1_ to S phase, upregulated cyclin D1, increased AKT and GSK3β phosphorylation, and decreased p16 and p21 expression. Additionally, activation of the AKT/GSK3β pathway induced by ectopic expression of PBX1 in HF-MSCs increased the translocation of β-catenin from the cytoplasm to the nucleus. We also showed that low expression of PBX1 inhibited cell proliferation and that the inhibitory activity of the AKT/GSK3β pathway abolished the effects of PBX1 overexpression. Collectively, our findings demonstrated, for the first time, that PBX1 enhanced HF-MSC proliferation through activation of the AKT/GSK signaling pathway.

As a potential TF involved in maintaining pluripotency, PBX1 was actively expressed in hESCs and HF-iPSCs. Our results indicated that *PBX1* expression was increased throughout the reprogramming process. Moreover, HF-iPSCs induced by SOMKP developed teratomas, which contained ectoderm, mesoderm, and endoderm, in immune-incompetent mice, suggesting that ectopic expression of PBX1 did not affect the totipotency and proliferation capacities of the cells. Compared with SOMK, SOMKP transduction significantly increased HF-iPSC colony formation and upregulated pluripotent gene expression. Additionally, the expression of pluripotency-related genes in SOMKP-transduced HF-iPSCs was significantly higher than that in hESCs-X0. Equivalence to ES cell lines is unlikely to be a sufficient indicator of an iPS cell line’s utility for a specific application, but it indicates the remarkable contribution of PBX1 to iPSCs reprogramming and maintenance of cell pluripotency. Further analysis revealed that during the early stages of reprogramming, PBX1 overexpression decreased the percentage of cells in early apoptosis by activating the AKT/GSK3β pathway and reducing the expression of apoptosis-related proteins. During the late stages of reprogramming, PBX1 greatly upregulated NANOG by activating the *NANOG* promoter, consistent with previous studies in hESCs [14]. Furthermore, PBX1 upregulated *NANOG* not only by activating the promoter but also by increasing the phosphorylation of AKT. Additionally, the inhibition of PBX1 expression and the AKT/GSK3β pathway increased the percentage of cells in early apoptosis during reprogramming and significantly decreased the generation of iPSCs. Taken together, these results suggested that PBX1 not only enhanced the generation of HF-iPSCs without blocking the induced pluripotency but was also essential for reprogramming.

In our previous study, AKT signaling was found to be essential for maintaining HF-MSC proliferation by upregulating cyclin D1 and downregulating p16 and p21 [18]. During reprogramming, activators of AKT also improve reprogramming efficiency. Studies of hiPSCs have demonstrated that increased phosphorylation of AKT and GSK3β induced by the inhibition of PHLDA3 expression enhances somatic cell reprogramming [8]. Similarly, by adding a small molecule activator of PDK1 to activate the downstream AKT, reprogramming efficiency is further enhanced [20]. This may occur through direct phosphorylation of GSK3β and subsequent phosphorylation of β-catenin by GSK3β. Moreover, phosphorylation of GSK3β at serine 9 promotes cell survival by inhibiting apoptosis [35, 36]. Additionally, our study suggested that β-catenin translocation caused by the activation of the AKT/GSK3β pathway in the presence of ectopic PBX1 expression may promote reprogramming efficiency. During reprogramming, β-catenin acts via interactions with telomerase reverse transcriptase/Brahma-related gene-1 and altered the structure of nucleosomes, thereby facilitating the binding of TFs and proteins to DNA [37, 38]. Additionally, we found that the knockdown of PBX1 or inhibition of AKT/GSK3β promoted the mitochondrion-mediated apoptotic cascade. In previous studies, p53 was found to block reprogramming in numerous cell lines. Inhibition of p53 expression or the expression of its target gene p21 improves reprogramming efficiency by decreasing the number of suboptimal cells via p53-dependent apoptosis [39, 40]. Our data suggested that p53 was downregulated by the AKT/GSK3β pathway. Western blot analysis showed that p53 mediated apoptosis by downregulation of the anti-apoptotic protein BCL2 and increased the expression of BAX. Moreover, the knockdown of PBX1 or inhibition of the AKT/GSK3β pathway regulated the activation of the p53 pathway, possibly inducing the translocation of stabilized p53 to the mitochondria, where p53 can directly interact with anti-apoptotic BCL2 and BAX [41]. BCL2 is localized in the outer wall of the mitochondria and acts to maintain membrane integrity and inhibit the release of cytochrome C. BAX is expressed in the cytosol but can translocate to the mitochondria and promote the release of cytochrome C [35]. Additionally, the translocation of cytochrome C promotes the cleavage of pro-caspase3 to caspase3, further accelerating the cleavage of PARP1, which is involved in DNA repair and chromatin remodeling. During the final step of apoptosis, PARP1 acts as a marker of apoptosis after cleavage by caspases [42]. Recent studies have indicated that increased PARP1 expression is detected throughout the reprogramming process and is involved in the efficient generation of iPSCs via PARP1-mediated epigenetic modulation and activation of pluripotency-related genes during reprogramming [43]. The coordination between PARP1 and TET2 promotes histone modifications; regulates the expression of SOX2, OCT4, and NANOG; and modulates chromatin structure during the reprogramming process [43, 44]. In our study, PBX1 knockdown or AKT/GSK3β inhibition reduced PARP1 expression and may have resulted in low reprogramming efficiency.

Dual-luciferase assays showed that PBX1 activated *NANOG* promoter activity. These findings were confirmed by qPCR, which demonstrated that PBX1 upregulated NANOG expression during the reprogramming process. NANOG plays a central role in maintaining the pluripotent state of stem cells and in the reprogramming of somatic cells into iPSCs. Both transforming growth factor β/activin and bFGF signaling pathways promote hiPSC and hESC pluripotency by sustainably maintaining the NANOG expression. In cooperation with OCT4, SOX2, and regulatory feedback loops, NANOG maintains the self-renewal and pluripotency of hiPSCs and hESCs [45–47]. Our results suggested that SOMKP induced iPSCs with a more active transcriptional network. Moreover, PBX1 has been reported to enhance the expression of pluripotency-related genes in hESCs [14]. Consistent with this report, we found that SOMKP transduction significantly regulated *ESG1*, *LIN28*, *NANOG*, endogenous *SOX2*, and *OCT4* expression during iPSC reprogramming. NANOG, OCT4, and SOX2 regulate their own promoters and other diverse pluripotency-related genes to form an extensive regulatory circuitry to maintain the pluripotency of hESCs and hiPSCs [48]. Additionally, pluripotency-related genes are downregulated during the differentiation of hiPSCs and hESCs in vitro [49]. Similarly, PBX1 was found to be expressed in undifferentiated hESCs and downregulated in differentiated cells [14]. As an upstream regulator of NANOG, overexpression of PBX1 enhances and maintains the high expression of pluripotency-related genes, probably potentially providing a significant route for maintenance of the pluripotency of HF-iPSCs in vitro. In the cell regulatory network, PBX1 prebound to the promoters of its target genes and subsequently interacted with other TFs to cooperatively activate the transcription of target genes [11, 50]. Similar to KLF4, OCT4, and SOX2, PBX1 could penetrate silent chromatin and bind to regulatory regions to increase DNA access for other proteins and active reprogramming at times when the overall chromatin structure still prevents access of other TFs [51, 52]. However, we discovered that NANOG expression was also regulated by the AKT/GSK3β pathway. Recent studies have revealed that activation of the Wnt/β-catenin pathway by inhibition of GSK3β results in β-catenin accumulation, which can help to maintain the self-renewal capacity of MSCs and hESCs by increasing NANOG expression [53]. Therefore, we concluded that PBX1 upregulated the NANOG expression to activate the *NANOG* promoter and increase the phosphorylation of AKT.

## Conclusions

In this study, we identified PBX1 as an important TF in enhancing HF-MSC proliferation and reprogramming, potentially by increasing AKT phosphorylation and β-catenin nuclear translocation. In HF-MSC reprogramming, PBX1 activated the *NANOG* promoter and upregulated NANOG expression. Moreover, PBX1 activated the AKT/GSK3β signaling pathway, inhibited mitochondrion-mediated apoptosis during the early stage of reprogramming, and upregulated endogenous *SOX2* and *OCT4* expression during the later stage of reprogramming. These results established a strategy for a large-scale acquisition of HF-MSCs and efficient generation of HF-iPSCs, which may have applications in regenerative medicine.

## Data Availability

All data generated or analyzed during this study are included in this published article.

## Abbreviations

HF: Hair follicle
MSC: Mesenchymal stem cell;
HF-MSCs: Human hair follicle mesenchymal stem cells
iPSCs: Induced pluripotent stem cells
HF-iPSCs: Human hair follicle mesenchymal stem cell-derived induced pluripotent stem cells
ESCs: Embryonic stem cells
HF-MSCs^EGFP^: HF-MSCs transfected with vector lentivirus
HF-MSCs^PBX1^: HF-MSCs transfected with lentivirus encoding *PBX1*
HF-MSCs^shRNA^: HF-MSCs transfected with lentivirus encoding *PBX1* short hairpin RNA
HF-MSCs^scrambled^: HF-MSCs transfected with scrambled lentivirus
OCT4: Octamer-binding transcription factor 4
KLF4: Kruppel-like factor 4
SOX2: SRY-box 2
SOKM: SOX2-OCT4-KLF4-cMYC
SOKMP: SOX2-OCT4-KLF4-cMYC-PBX1
PBS: Phosphate-buffered saline
SSEA: Stage-specific embryonic antigen
qPCR: Quantitative polymerase chain reaction
bFGF: Basic fibroblast growth factor
CDK: Cyclin-dependent kinase
PI: Proliferation index
TFs: Transcription factors
APC: Allophycocyanin
DMEM: Dulbecco’s modified Eagle’s medium
7AAD: 7-Amino actinomycin
DMSO: Dimethyl sulfoxide
FBS: Fetal bovine serum
PARP: Poly (ADP ribose) polymerase 1
shRNA: Short hairpin RNA
H&E: Hematoxylin and eosin
